# Liver disease in adults with severe alpha-1-antitrypsin deficiency

**DOI:** 10.1007/s00535-019-01548-y

**Published:** 2019-01-24

**Authors:** Hanan A. Tanash, Eeva Piitulainen

**Affiliations:** Department of Respiratory Medicine and Allergology Malmö, Skåne University Hospital, Lund University, Jan Waldenströms gata 24, plan 4, 205 02 Malmö, Sweden

**Keywords:** Alpha-1-antitrypsin deficiency, Liver disease, Liver function tests

## Abstract

**Background:**

The proportion of adults with liver disease due to severe alpha-1-antitrypsin deficiency (AATD), with PiZZ phenotype, is not clear. The markers of the AATD liver disease, how it progresses, and measures for its prevention have not been established. The aim of this study was to analyze the risk of liver disease in individuals with severe AAT deficiency (PiZZ).

**Methods:**

Longitudinal clinical and laboratory data were obtained from the Swedish National registers, by cross-linkage between the Swedish national AATD register, the Swedish National Patient Register, the National Cancer Register and the National Causes of Death Register.

**Results:**

A total of 1595 PiZZ individuals were included in the analyses. The mean follow-up time was 12 years (range 0.3–24). The mean number of follow-ups was 5 (range 2–15). Two or more liver function tests (LFTs) were available in 1123 individuals, and 26% of them (*n* = 290) had repeated elevated LFTs during the follow-up. The prevalence of any liver disease was 10% (*n* = 155). Liver cirrhosis was found in 7% of the individuals (*n* = 116) and hepatocellular carcinoma in 2% (*n* = 29). The mean age at the onset of liver disease was 61 (SD 15) years. In multivariate analyses, the male gender, age over 50 years, repeated elevated LFTs, hepatitis virus infection, and a diagnosis of diabetes were associated with increased risk of developing liver disease in adulthood (*p* < 0.01).

**Conclusion:**

The prevalence of liver disease in adult PiZZ individuals is 10%. Age over 50 years, the male gender, repeated elevated liver enzymes, hepatitis, and the presence of diabetes mellitus are risk factors for developing liver disease.

## Introduction

Severe alpha-1-antitrypsin deficiency (PiZZ) is a well-known risk factor for developing chronic obstructive pulmonary disease (COPD) and liver disease. Pathogenesis in the liver disease is due to the accumulation of alpha-1-antitrypsin (AAT) in the rough endoplasmic reticulum through a mechanism of polymerization. Polymerization is favored by the incorrect tertiary structure of the AAT molecule caused by a point mutation at position 342 of the protein [1–3]. The accumulation of AAT in the liver cells is not sufficient per se to explain the liver disease which is not manifested in all PiZZ subjects and thus, a trigger factor must be hypothesized.

Although the PiZZ genotype is defined by a single gene defect, the presentation of liver disease is highly variable. Children may present neonatal cholestasis with jaundice, which often reverts to normal but may also progress to cirrhosis [4]. In adults, liver disease is often undetected until cirrhosis or hepatocellular carcinoma is evident. The American Thoracic Society/European Respiratory Society recommend a regular assessment by simple liver function tests (LFTs) in PiZZ individuals [1]. However, it is unclear whether these liver function tests can be used to predict or identify liver disease.

The prevalence of liver disease and its natural history in PiZZ individuals have been difficult to assess. Furthermore, factors potentially contributing to the progression of liver disease in severe AAT deficiency (AATD) have been poorly studied. Previously published data have indicated that in PiZZ individuals about 20% of the causes of death are liver disease, i.e., liver cirrhosis or hepatocellular cancer [5]. Therefore, the early identification of PiZZ individuals who are at risk of developing liver disease is of importance. The aim of this study was to analyze the risk of liver disease in individuals with severe AAT deficiency included in the Swedish national AATD register.

## Methods

Clinical and laboratory data were obtained from the National Swedish registers, by cross-linkage between the Swedish national AATD register, the Swedish National Patient Register, the National Cancer Register and the National Causes of Death Register. These Registers were cross-linked using the Swedish system of personal identity numbers.

### Study population and data collection

The PiZZ subjects were identified from the Swedish National AATD Register, which has been described in detail elsewhere [6]. Eligibility criteria for enrolment in the register are age ≥ 18 years, severe AAT deficiency (PiZZ, PiZNull and PiNullNull) and written, informed consent. Clinical examination, blood samples (liver function tests) and results of lung function tests are performed at the patient’s local hospital. Thereafter, the results are reported to the AATD register every 2 years via a questionnaire, which is answered by the attending physician. More than 95% of the PiZZ individuals who are identified in Sweden have agreed to be included in the AATD register.

The indications for plasma protein analysis leading to PiZZ diagnosis were the following: respiratory diseases or symptoms, including repeated respiratory tract infections; liver diseases or elevated liver function tests, other diseases and family/population screening. Other diseases included renal diseases, joint symptoms, repeated infections other than respiratory tract infection, high sedimentation rate, or other signs and symptoms for which plasma protein analysis was performed as part of the clinical investigation.

### Clinical data

Data on diagnoses of liver disease and procedures including surgical and medical procedures during the follow-up time were obtained from the Swedish National Patient Register (SNPR) and the National Cancer Register [7, 8]. The SNPR covers more than 99% of all the hospitalizations since 1987 and about 80% of all the hospital-based outpatient care since 2001 nationwide [7]. Diagnoses were coded according to the 9th (before 1996) and the 10th revisions of the WHO International Classification of Disease (ICD). ICD codes were grouped (ICD-9; ICD-10) as liver disease (570–573; K70–K76), liver failure (570; K72), hepatitis (573D; K73), liver fibrosis (K740, K742), cirrhosis (571F, C, X; K746), biliary cirrhosis (571G; K743, K745), fatty liver (573; K760), hepatocellular cancer (155–156; C22–24), chronic hepatitis B (070.3; B181), chronic hepatitis C (070.7; B182), panniculitis (729.3; M793), COPD (490–492, 496; J40–J44), hypertension (401–405; I109; I12–I15), and diabetes (250; E10–E15). Liver disease was assessed through a two-step process. First, all the ICD codes (diagnoses, complications, and procedures) in the SNPR and the National Cancer Register were screened and categorized by a data manager. Second, the study database was examined by HT who manually evaluated all the ICD codes. The assessment and categorization were blinded to all the patient covariates.

Vital status was obtained from the National Causes of Death Register. Patients were followed prospectively from the date of inclusion in the register (baseline) until the date of death, or study end (August 15, 2015).

### Liver function tests

The following laboratory analyses were performed at the patient’s local hospital and reported to the registry every 2 years: plasma aspartate aminotransferase (AST) with a reference interval for women of 14.9–35.9  U/L and for men 14.9–44.9  U/L; plasma alanine aminotransferase (ALT) with a reference interval for women of 9.0–44.9  U/L and for men 9.0–65.9  U/L; plasma alkaline phosphatase (ALP) with a reference interval of 35.9–107.8  U/L; plasma gamma-glutamyl transpeptidase (GGT) with a reference interval for women of 9.0–44.9  U/L and for men 9.0–113.8  U/L. The time interval between the LFTs was 1–4 years. The majority of individuals had at least two tests during the follow-up. The results of the LFTs at inclusion and during follow-up were analyzed. The individuals who had repeated (at two or more follow-ups) elevation of two or more liver enzymes were classified as individuals with repeated elevated liver function tests.

### Ethical considerations

The study was approved by the Lund University Research Ethics Committee (2015/186). In accordance with Swedish research regulations for register-based research, individual patient consent on being included in the study was not required.

### Statistical methods

Baseline data were tabulated using frequencies and percentages for categorical variables, mean with SD. Continuous variables with normal distribution were compared using ANOVA.

Patients were followed prospectively from the date of inclusion in the register (baseline) until the date of liver disease, date of death, or study end (August 15, 2015). For the analysis of survival of patients with liver disease, patients were followed from the date of liver disease onset to the date of the first of the following events: liver transplantation, death, or study end. Cumulative crude survival probabilities were estimated using the Kaplan–Meier method and life tables. The Cox analysis was performed to predict the risk factors for developing liver disease, using the newly diagnosed liver diseases (during follow-up) as events. The model was adjusted for baseline age, gender and smoking status. The time-dependent covariates to be adjusted for were repeated elevated LFTs, hepatitis infection and the presence of any of the following comorbidities during the follow-up time: COPD, hypertension and diabetes.

## Results

A total of 1595 PiZZ individuals were included in the analyses. The demographic data are presented in Table 1. The Pi-phenotype was available in all the cases. The mean follow-up time was 12 years (range 0.3–24). The mean number of follow-ups was 5 (range 2–15).

**Table 1:** Demographic data of 1595 PiZZ individuals at inclusion in the register

Variable	
Men, *n* (%)	781 (49)
Age	
At diagnosis of AATD	41 (21)
At inclusion	47 (17)
Basis for identification	
Respiratory diseases	678 (43)
Screening	375 (24)
Liver	112 (7)
Other	420 (26)
Smoking habits	
Ever-smokers	862 (54)
Never-smokers	733 (46)
Lung function	
FEV_1_, % of predicted	74 (33)
FVC, % of predicted	84 (23)
FEV_1_/FVC ratio	0.64 (22)
Liver function test, U/L	
AST (men)	33.5 (16.8)
AST (women)	26.4 (12.0)
ALT (men)	38.3 (26.9)
ALT (women)	24.0 (16.8)
GGT (men)	53.9 (70.6)
GGT (women)	37.7 (51.50)
ALP	143.7 (76.1)

Data presented as mean (standard deviation) or frequency (percentage)

*AATD* severe AAT deficiency, *FEV*_*1*_ forced expired volume in 1 s, *FVC* forced vital capacity

### Liver diseases

The prevalence of liver diseases at identification of AAT deficiency, at inclusion in the register, and at the end of the study is summarized in Table 2. The total number of subjects with any liver disease was 10% (*n* = 155). Liver cirrhosis was found in 7% of the (*n* = 116) individuals. Of these, 13 had malignant transformations. Hepatocellular carcinoma was found in 2% (*n* = 29). A liver biopsy was done in 33 cases. The mean age at the onset of liver disease was 61 (SD 15) years. In 124 patients (80%), the onset of liver disease was at the age of 50 years or older. Men were significantly more likely to have liver disease than women (*p* < 0.001) (Table 3).

**Table 2:** Prevalence and occurrence of liver disease at diagnosis of AAT deficiency, at inclusion in the AATD register, and at the closing point of the study

Liver disease	At identification (*N* = 55)	At inclusion (*N* = 53)	At study end (*N* = 155)
Liver cirrhosis	45	49^a^	103
Fatty liver	4	2	17
Unspecific hepatitis	2	Recovered	6
Hepatocellular carcinoma	2	2	29^b^
Neonatal cholestasis	2	Recovered	–

^a^Includes 2 patients with fatty liver who developed liver cirrhosis before inclusion in the register

^b^Includes 13 patients with liver cirrhosis who developed hepatocellular carcinoma during the follow-up

**Table 3:** The results for the PiZZ individuals with the diagnosis of liver disease and those without

Variable	Liver disease (*N* = 155)	No liver disease (*N* = 1440)	*p* value
Men	100 (65)	681 (47)	0.000
Age at inclusion	54 (17)	46 (16)	0.000
Basis of identification			0.000
Hepatic disease	53 (34)	59 (4)	
Non-hepatic	102 (66)	1381 (96)	
Respiratory symptoms	107 (69)	883 (61)	0.06
Ever-smokers	87 (56)	775 (54)	0.61
Pack years	16 (12)	14 (13)	0.16
FEV_1_ (% of predicted value)	68 (31)	74 (33)	0.04
FVC (% of predicted value)	81 (20)	84 (23)	0.09
COPD	63 (41)	467 (32)	0.04
Liver function test at baseline			
AST (men)	48.5 (30.5)	31.7 (12.6)	0.000
AST (women)	35.9 (22.7)	24.6 (9.6)	0.000
ALT (men)	59.3 (47.9)	34.7 (23.9)	0.000
ALT (women)	31.7 (17.9)	25.2 (19.2)	0.057
GGT (men)	104.8 (121.0)	44.9 (52.7)	0.000
GGT (women)	65.9 (70.6)	35.3 (47.9)	0.005
ALP	183.2 (123.3)	139.52 (68.9)	0.000
During the follow-up			
Repeated elevated LFT during follow-up^a^	112 (72)	178 (12)	0.000
Hepatitis C and B infections	10 (6)	6 (0.4)	0.000
COPD	32 (21)	281 (20)	0.44
Hypertension	42 (27)	258 (18)	0.01
Diabetes	35 (23)	86 (6)	0.000

Data presented as mean (standard deviation) or frequency (percentage)

^a^Two or more elevated tests for two or more follow-ups

Liver dysfunction as the basis for identification was reported for 112 subjects (7%). Of them, 55 also had a diagnosis of liver disease, and 57 only had occasionally elevated liver function tests. None of these subjects with occasionally elevated liver function tests at identification developed any liver disease during the follow-up. Two of the four patients with fatty liver at diagnosis developed liver cirrhosis before their inclusion in the register (Table 2).

At inclusion in the register, 53 patients (3%) manifested liver diseases, and during the follow-up time, an additional 102 individuals (7%) developed liver diseases. At the closing point of the study, 103 subjects (7%) had liver cirrhosis, and 29 had HCC (2%, including those 13 with malignant transformation of cirrhosis), as shown in Table 2.

### Liver function tests

LFTs were available in 88% of the study population at baseline; 88% in patients with liver disease and 85% in those without liver disease. Two or more LFTs were available in 1123 (70%) individuals.

The results of the LFT in the subjects with and without liver disease are shown in Table 3. The mean AST, GGT and ALP at baseline were significantly higher in the subjects with liver disease (*p* < 0.01), except for the mean ALT that did not differ significantly between the women with and those without liver disease.

During the follow-up time, the frequency of repeated elevated LFTs was significantly higher in the subjects with liver disease compared with the group without. The LFTs were elevated 2–4 years prior to diagnosis of liver disease in 108 (70%) individuals who developed liver disease during the follow-up.

### Liver transplantation

Eight patients with liver cirrhosis underwent liver transplantation during the follow-up. One of them died of colon carcinoma 15 months after the transplantation.

### Other diseases

A total of 843 (53%) PiZZ individuals had the diagnosis of COPD. Of these, 313 (37%) developed COPD during the follow-up. Fifty-four percent (*n* = 862) were ever-smokers, with a mean number of pack years of 14 (SD 12). No differences in tobacco consumption and smoking habits were found between the subjects with and those without liver disease, which is shown in Table 3. The proportion of subjects with COPD at inclusion was higher among the individuals with liver disease than those without liver disease. Chronic hepatitis viral infection was found in 16 patients (3 with hepatitis B and 13 with hepatitis C virus), which is shown in Table 3. The diagnosis of hepatitis C infection was significantly higher in patients with liver disease (*n* = 8; 5%) compared to those without liver disease (*n* = 5; 0.3%).

Four patients had the diagnosis of panniculitis. None of them developed liver disease. The presence of diabetes and hypertension was higher in subjects with than those without liver disease. Patients who developed liver disease were older, included a higher proportion of men, and had a lower FEV_1_ and higher LFTs at inclusion and during the follow-up, as shown in Table 3. BMI was not included in the analysis, because it was available only in 524 subjects (33%).

### Risk factors for liver disease

A multivariate analysis was performed to assess the risk factors for developing liver disease and demonstrated that the male gender, age over 50 years, hepatitis infection, and the presence of repeated elevated LFTs were risk factors for liver disease in adulthood. Smoking habits and the development of hypertension were not associated with any increased risk for liver disease. The development of diabetes and COPD during the follow-up predicted independently the occurrence of liver disease (Table 4).

**Table 4:** Multivariate analysis for the risk of liver disease development for the PiZZ individuals

Variables	Multivariate analysis	
	RR (95% CI)	*p* value
Male gender	1.45 (1.15–2.14)	0.03
Age ≥ 50 years	2.02 (1.30–3.16)	0.002
Ever-smokers	0.85 (0.56–1.30)	0.46
Repeated elevated LFTs	7.66 (5.10–11.73)	0.000
Hepatitis infection	3.12 (1.21–8.08)	0.02
COPD	2.20 (1.32–3.70)	0.003
Hypertension	0.91 (0.50–1.66)	0.76
Diabetes	3.87 (2.18–6.87)	0.000

### Survival and causes of death

One hundred and six (68%) of the 155 subjects with liver disease died during the follow-up. The median age at death was 68 years (range 24–88). The estimated median survival time after the onset of liver disease was 1.8 years (95% CI 0.73–2.95). The 1-year probability of survival after the onset of liver disease was 55%.

The causes of death were liver failure in 53 patients; hepatocellular cancer in 29, cardiovascular disease in 6 patients, gastrointestinal bleeding in 6 patients, peritoneal abscess in 3 patients, respiratory failure in 5 patients, gastrointestinal cancer in 2 patients, septicemia in one patient, and traffic accident in one patient.

## Discussion

Our study is the largest existing follow-up of PiZZ individuals, and shows that the prevalence of liver disease in adult PiZZ individuals is 10%. Age over 50 years, male gender, repeated elevated liver enzymes, hepatitis virus infection, the presence of diabetes mellitus and COPD are risk factors associated with the development of liver disease.

Knowledge of the proportion of adults who develop liver disease because of severe AATD has been poor despite several studies have assessed the prevalence of liver disease in PiZZ individuals [9–15]. One of the widely studied effects of AATD on the liver is reported in the Swedish study published by Eriksson [9]. He analyzed the results of all the post-mortem examinations in Malmö, Sweden and found that 43% of PiZZ individuals had cirrhosis, and 28% had hepatocellular carcinoma (HCC). Furthermore 5–10% of the PiZZ individuals over 50 years of age had developed cirrhosis. Massi et al. have reported that the most frequent histologic picture in liver disease is cirrhosis that quickly evolves into liver failure and death [14]. He has also reported that cirrhosis accelerates by the impaired repair of hepatic connective tissue structures damaged by inflammatory proteases, and that the destruction of the liver escalates when onset occurs in PiZZ adults. Liver cancer usually develops in a patient with cirrhosis or other chronic liver disease. Occasionally, hepatocellular carcinoma (HCC) occurs in PiZZ patients without preceding cirrhosis [14]. In our study, liver cirrhosis was found in 7%, and hepatocellular cancer in 2%. Almost half (13) of the 29 patients with HCC had malignancy transformation from liver cirrhosis. However, the majority (16 patients) had HCC as a primary diagnosis. Furthermore, liver disease was at an advanced stage at the time of diagnosis, with a short median survival time of less than 2 years after the diagnosis, and with a 1-year probability of survival of only 55%. In addition, other similar results have been published previously showing that PiZZ subjects with liver disease are prone to develop HCC [9, 10, 16]. In addition, our previously published study has shown an increased risk of mortality due to liver cirrhosis and hepatocellular carcinoma in PiZZ individuals [17].

Although severe AATD is considered as a significant risk factor for the development of cirrhosis in adulthood, there is limited information on other risk factors contributing to it. The markers of the AATD liver disease, factors that influence its progression and prevention measures are not known. In our study, the frequency of viral hepatitis C was significantly higher among the patients with liver disease compared with those without. Hepatitis infection was also an independent risk factor for the occurrence of liver disease. A similar finding has been reported by Vogel et al. in a study that showed that hepatitis C virus was found frequently in patients with liver disease and AATD (mild and severe deficiency). Therefore, hepatitis C virus is a possible trigger factor for liver disease [15]. In contrast, Bowlus et al. have not found alcohol consumption and viral hepatitis to be associated with increased risk of liver disease in AAT-deficient adults, but male gender and obesity could predispose to advanced liver disease [13]. Therefore, further studies are necessary to elucidate this aspect.

An interesting finding in our study was the association of diabetes mellitus with the increased risk of developing liver disease in PiZZ individuals. Diabetes and insulin resistances have been reported to be independent risk factors for liver fibrosis and cirrhosis [18–21]. Even the risk of developing hepatocellular carcinoma may be increased in patients with diabetes [22, 23]. A large population-based, matched retrospective cohort study using the administrative health databases from the province of Ontario, Canada, has reported that adults with newly diagnosed diabetes are at a significantly higher risk of developing serious liver disease (liver cirrhosis, liver failure and its sequelae, or receipt of a liver transplant) than those without diabetes [21]. This study suggested that diabetes, with or without preexisting hypertension, dyslipidemia or obesity, conferred a higher risk of serious liver disease than any of the three other conditions in isolation. The authors posited that the presence of overt diabetes reflects severe insulin resistance, a greater fatty load in the liver and potentially worse hepatic inflammation and injury. However, it is unclear whether diabetes is more harmful to the liver in individuals with severe AATD or whether the hepatocyte is more susceptible to glucotoxicity than in subjects with normal AAT levels. More studies are needed in this field [21].

Neonatal hepatitis is rarely noted in the medical history of adult PiZZ patients with liver disease [14]. We have recently analyzed liver function at 40 years of age in PiZZ subjects identified by neonatal screening, and found that none of the subjects with liver disease in the neonatal period who survived childhood suffered from liver disease in adulthood [24]. Furthermore, they had normal lung function in adulthood. Thus, non-fatal liver disease in childhood seems not to influence directly the risk of liver disease in adults up to 40 years of age.

We did not find any association between smoking habits and the risk of liver disease. Thus, the occurrence of liver disease seems to be independent of smoking habits. However, lung function was poorer in the subjects with liver disease, and COPD was more frequent among patients with liver disease compared with those without. When the occurrence of COPD during the follow-up was used as a time-dependent covariate in the COX analysis, its association with an increased risk of liver disease remained significant.

COPD is the most common diagnosis and cause of death in adult PiZZ individuals. Liver disease is less prevalent. Our recently published data on the cohort of AAT-deficient subjects identified by the Swedish national neonatal screening program in 1972–1974 have also shown that in PiZZ smokers, COPD occurs before the age of 40 years, but no signs of liver disease are present [24, 25]. Thus, it is possible that liver disease occurs later than COPD in adult PiZZ individuals, and many PiZZ smokers do not survive long enough to develop liver disease. By decreasing smoking frequency, the prevalence of liver disease may increase in PiZZ individuals.

Liver function tests were available for the majority of the PiZZ subjects at inclusion in the register and during the long follow-up time, giving us the opportunity to determine whether repeated LFTs are helpful for the detection and prediction of liver disease. LFTs at inclusion gave significantly higher results in the subjects with liver disease compared with those without. Repeated LFT abnormalities were found in 70% of the individuals with liver disease 2–4 years prior to diagnosis of liver disease. On the other hand, occasionally elevated LFTs at identification did not predict any liver disease. Therefore, repeated liver function tests may be helpful in detecting the early stage of liver disease in PiZZ adults. The American Thoracic Society/European Respiratory Society have recommended a regular assessment of simple liver function tests (LFTs) in the PiZZ individuals who were asymptomatic or had only lung disease [1].

## Clinical application

Regular monitoring of liver function by laboratory tests in PiZZ subjects is of importance for the early detection of liver disease. However, due to the high prevalence of liver cirrhosis and hepatocellular cancer, imaging technics by ultra sound may improve the early detection of liver disease in PiZZ individuals.

## Strengths and limitations

Sweden offers a unique opportunity to study the risk of disease in individuals with AATD by cross-linkage between the Swedish national AATD register, the Swedish National Patient Register, the National Cancer Register and the National Causes of Death Register, which have a near complete coverage and follow-up.

The Pi phenotyping is performed at one central laboratory. The laboratory reports personal data of all newly diagnosed PiZZ subjects to the AATD register. Thus, more than 95% of all the adult PiZZ individuals are included in the register. The prevalence of the PiZZ phenotype being 1/1600 in Sweden, about 25% of all the adult PiZZ individuals are included in the register.

The follow-up time was long, up to 24 years since 1991, when the AATD register was started. Repeated analyses of liver enzymes have allowed longitudinal follow-up of liver function and occurrence of liver disease in the study participants.

The most important limitation is the fact that no information about alcohol use among the individuals included in the register was available. Our analyses also lack data on medication, which may influence the liver function tests. BMI was not included in the analyses because it was available only for a limited number of the subjects (33%). Furthermore, a register-based study can never be regarded as a real epidemiological study.

## Conclusion

The prevalence of liver disease in adult PiZZ individuals is relatively high. Regular and repeated monitoring of liver function by laboratory tests in PiZZ subjects is of importance for the early detection of liver disease.
